# Genitourinary rhabdomyosarcoma in a child with urinary incontinence as the initial symptom: A case report and literature review

**DOI:** 10.1016/j.eucr.2025.103051

**Published:** 2025-04-23

**Authors:** Jingde Wu, Rong Ma, Qingwei Zhang, Jianlin Xie, Xiande Huang

**Affiliations:** a The First Clinical Medical College of Gansu University of Chinese Medicine (Gansu Provincial Hospital), Lanzhou, China; b Department of Urology, Gansu Provincial Hospital, Lanzhou, China

**Keywords:** Rhabdomyosarcoma, Genitourinary system, Pediatric urinary incontinence

## Abstract

Rhabdomyosarcoma (RMS) is a morphologically heterogeneous high-grade malignancy that occurs uncommonly in the pediatric genitourinary (GU) system, and the most typical clinical symptoms are urinary frequency, urgency, hematuria, and associated with infection. The boys in this case, persistent urinary incontinence was relatively rare, and it was easily misdiagnosed as pediatric urinary incontinence in clinical diagnosis. Incontinence disease and lower urinary tract symptoms were identified by imaging examination, and final pathological biopsy confirmed the diagnosis of pediatric GU embryonal rhabdomyosarcoma (ERMS). Learning through case report and literature can help clinicians to better understand the manifestations and accurate diagnostic treatment of the disease.

## 1 Introduction

Rhabdomyosarcoma (RMS) is a morphologically and clinically heterogeneous high-grade malignant tumor thought to originate from skeletal muscle cell lineage. The cancer cells demonstrate a tendency toward myogenic differentiation,^1^ is the most common soft tissue sarcoma in children, with approximately half of all soft tissue sarcomas in pediatric patients being RMS, RMS of the pediatric genitourinary (GU) system, with urinary incontinence as the primary clinical symptom has rarely been reported in the previous literature. The authors report a case of urinary incontinence in a child as the first symptom with a final diagnosis of RMS.

## 2 Case report

**Fig. 1:**
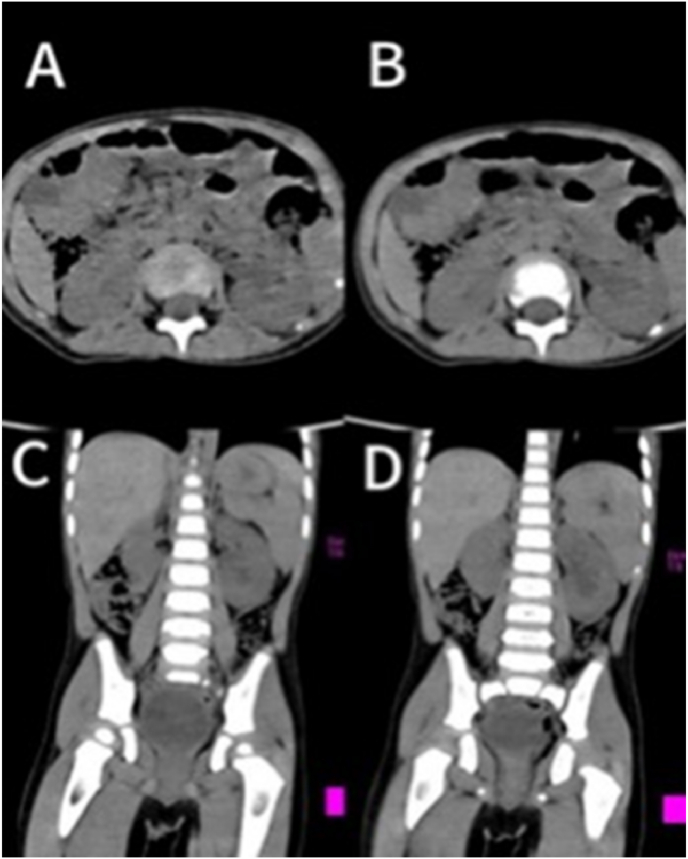
(A–D):plain CT scan of the urinary system revealed hydrodilation of the left renal pelvis and ureter. (A–B): Plain CT scan in the horizontal plane of the urinary system; (C–D): Plain CT scan in the coronal view of the urinary system.

**Fig. 2:**
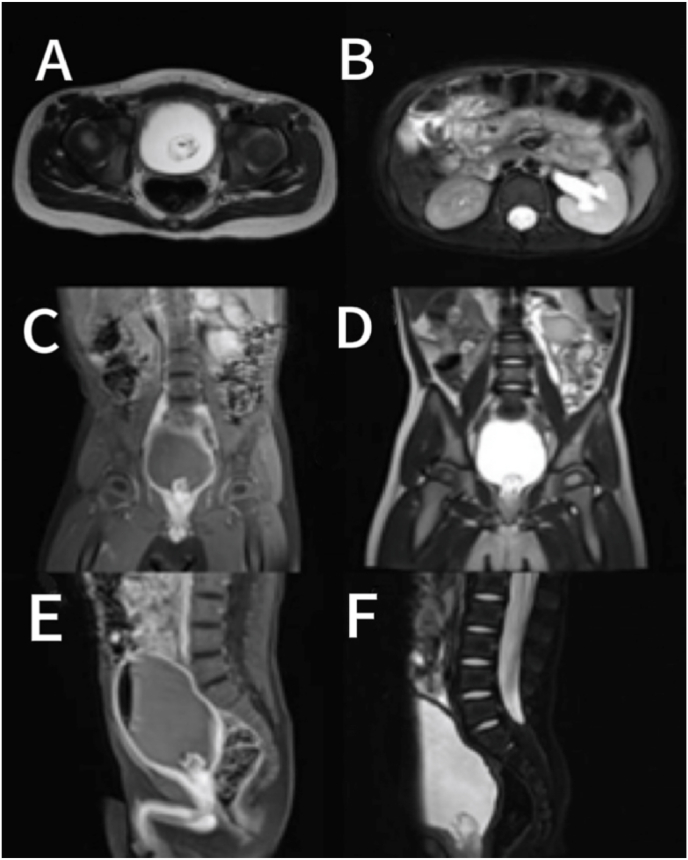
(A–F): Urinary plain scan MR, urinary enhanced MR. A: Urinary routine scan in MR T2W1 horizontal plane; B: Urinary enhanced MR T2W1 with lipid suppression technique; C: Urinary enhanced MR T1W1 in coronal orientation; D: Urinary routine scan in MR T2W1 coronal plane; E: Urinary enhanced MR T1W1 in sagittal view; F: Urinary enhanced MR T2W1 with fat saturation technique.

**Fig. 3:**
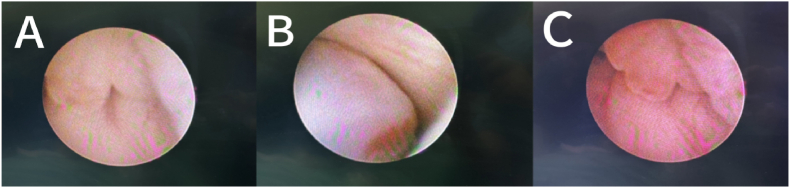
(A–C): Transurethral cystoscopy revealed a linear and hydrocyst -like mass measuring 1.5cm from the distal part of the verule at 5 o'clock position (near the urethral sphincter).

**Fig. 4:**
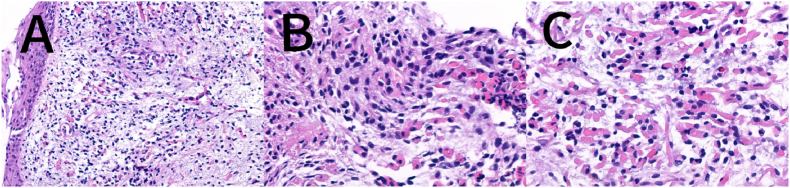
(A–C): Pathological examination immunohistochemistry: embryonal rhabdomyosarcoma.

A 2-year and 1-month-old boy presented with a chief complaint of “persistent involuntary urinary incontinence for over 1 month,” occasionally accompanied by pain during urination, bedwetting at night, No other special symptoms, medical history and physical examination were not special. Urological outpatient examination 25-hydroxyvitamin D: 37ng/ml (reference value:≥20ng/ml suggests vitamin D sufficiency, ≥100ng/ml suggests vitamin D poisoning), The Videourodynamic study revealed a bladder with low compliance and detrusor hyperactivity, measured residual urine 97ml.The urinary Ultrasound(UR) showed a hyperechoic mass measuring approximately 29 × 31mm in the bladder triangle, leading to an initial diagnosis of bladder mass. The urological plain CT scan showed dilated fluid in the left renal pelvis and ureter (Fig. 1). Lumbar plain MR scan results were not special, excluded spinal neurological lesions. The urinary plain scan combined with enhanced MR showed a 30.5 × 34.0 × 26.7mm mass in the bladder with clear borders, occupying the posterior urethra of the prostate. (Fig. 2). Our team completed a transurethral cystoscop, and found that the patient's posterior urethra was 1.5 cm from the distal end of the seminal colliculus, and a striated mass was visible in the 5 o'clock direction (approximately at the urethral sphincter), growing the posterior urethra towards the triangular area of the bladder neck and compresses the urethra (Fig. 3). Three sites of the tumor were taken for biopsy: mesenchymal tumor; Embryonal RMS (ERMS), (Fig. 4). Following multidisciplinary consultation and discussion, neoadjuvant chemotherapy comprising vincristine, actinomycin D, and cyclophosphamide (VAC) was administered.

## 3 Discussion

In recent years, the overall incidence of RMS in individuals under 20 years of age has been approximately 4.5 cases per million, with a lower incidence in Asia, with an average of 2 cases per million in the Japanese, Indian, and Chinese populations.^2^ RMS is more prevalent in males, with a male to female ratio of 1.37:1, for reasons that are unclear, the age of onset is mainly bimodal, with a peak between the ages of 2 and 6 years, followed by another peak between the ages of 10 and 18 years.^3^ The prevalence of GU RMS is approximately 20 %,^1^^,^^4^ specifically the bladder/prostate and the reproductive tract, which includes the female reproductive tract (vagina, uterus, cervix).^5^

From the pathological classification, this child was pathological classification as ERMS, which is also the most common type of RMS in children, accounting for about 60–70 %, which is the main subtype of the head, neck and genitourinary system in young children.^6^ GU RMS typical clinical symptoms include urinary frequency, urgency and hematuria, also with the possibility of infection. In severe cases, patients may present with abdominal distension, constipation, and urinary retention. Even urinary incontinence, but is often overlooked during clinical diagnosis. Occasionally patients will have signs of systemic malignancy, but PSA levels are usually normal.^7^, ^8^, ^9^UR is the most commonly used imaging technique for examining genitourinary masses and their adjacent organs, and is also the first choice for patients with voiding dysfunction. The evaluation of primary tumors should involve the use of enhanced scanning in both MRI and CT modalities, considering the potential risks associated with CT radiation exposure, MRI is recommended for assessing primary tumors located in the genitourinary system in pediatric patients. The primary tumor should be assessed for its dimensions and proximity to the surrounding anatomical structures, as well as for the presence of lymph node involvement and metastasis, which occurs in approximately one out of every six patients with RMS. The principal sites of metastatic spread include the lungs, liver, and bones.^10^^,^^11^

Treatment modalities for RMS include pathological biopsy, risk stratification, neoadjuvant chemotherapy, followed by local control through delayed primary excision surgery or radiotherapy, prioritizing organ-preservation approaches (*e.g.*, bladder preservation).^12^^,^^13^ The survival rates have shown significant improvement over the past two decades, with 5-year survival rates reaching 85 % in patients diagnosed with low-grade, localized ERMS.^4^ The initial procedure involved cystoureteroscopy and pathological biopsy, the tumor's gross appearance, immunohistochemical staining, and histopathology were also significant factors in reaching a conclusive diagnosis. In RMS cases, SMA, S-100, and vimentin showed positive results while cytokeratin was negative. Immunohistochemical markers such as Desmin, Myogenin, and MyoD1 exhibited high specificity and sensitivity for diagnosing RMS.^14^ The primary treatment is still chemotherapy, and rational chemotherapy regimens can improve the overall prognostic survival of patients with genitourinary RMS.^15^ The patient was classified as low-risk in the final assessment of risk, and the 3–6 month VAC regimen remains the primary recommendation for RMS chemotherapy in low-risk children.^16^

This child presented with persistent urinary incontinence as the initial symptom and was ultimately diagnosed with GU RMS, an exceptionally rare condition in clinical practice. Based on clinical experience, clinicians would initially consider congenital urinary tract anatomic malformation, external urethral sphincter dysfunction, or other iatrogenic causes when evaluating incontinence symptoms. Overactive bladder, delayed voiding, and functional voiding disorders being the most common causes. The simultaneous consideration of potential neurological lesions, such as brain injury, spinal cord dysplasia or spinal cord lesions in children, leads to misdiagnosis as pediatric urinary incontinence and mental frequency of urination. The organic lesions affecting the bladder and urethra during childhood development are frequently overlooked. Urinary incontinence resulting from spinal cord and spinal lesions was ruled out following the patient's admission to the hospital through imaging examination. Subsequently, a tumor located in the posterior urethra near the external urethral sphincter extending into the bladder was identified, and neoadjuvant chemotherapy was administered after biopsy confirmation of ERMS. In children presenting with lower urinary tract symptoms, particularly those with urination disorders and urinary incontinence, a comprehensive clinical evaluation is necessary. A detailed medical history, physical examination, laboratory tests, imaging studies, urethral function assessment and exclusion of potential underlying pathologies such as bladder or prostate neoplasms in order to avoid late diagnosis and delayed treatment.

## Funding

This work was supported by the Research Foundation of Gansu Provincial People's Hospital (grant number 23GSSYD-14) and by the Collaborative Project of the 10.13039/501100012166National Key Research and Development Program of China (grant number HX-62000001-2025-004).

## CRediT authorship contribution statement

**Jingde Wu:** Writing – original draft, Investigation, Formal analysis, Conceptualization. **Rong Ma:** Resources, Funding acquisition. **Qingwei Zhang:** Visualization, Validation, Resources. **Jianlin Xie:** Resources, Investigation, Conceptualization. **Xiande Huang:** Writing – review & editing, Validation, Supervision, Funding acquisition.

## Conflicts of interest

None.
